# Left Ventricular Hypertrabeculation: Historical Evolution, Diagnostic Pitfalls, and a Pragmatic Clinical Approach

**DOI:** 10.31083/RCM51235

**Published:** 2026-05-22

**Authors:** Alessandro Parodi, Marika Martini, Annagrazia Cecere, Lorena Iezzi, Ilaria Rigato, Barbara Bauce

**Affiliations:** ^1^Department of Cardio-Thoraco-Vascular Sciences and Public Health, University of Padua Medical School, 35128 Padua, Italy; ^2^Department of Neuroscience, Imaging and Clinical Sciences, G. D’Annunzio University of Chieti-Pescara, 66100 Chieti, Italy

**Keywords:** left ventricular hypertrabeculation, left ventricular non-compaction, hypertrabeculation, cardiomyopathy, phenotypes, management

## Abstract

Left ventricular hypertrabeculation (LVHT), previously referred to as left ventricular non-compaction (LVNC), has experienced fluctuating recognition in cardiology. Once defined as a primary genetic cardiomyopathy, this intriguing myocardial feature is now considered a “trait” in the most recent guidelines. However, the understanding of this phenomenon remains incomplete. Moreover, data on the association between this feature and other syndromes are inconclusive, and further advances in understanding the associated molecular mechanisms and genetic background are needed. A systematic collection of clinical data is essential to avoid both over- and underdiagnosis, thereby reducing current uncertainties in LVHT management. This review examines the diagnostic, pathophysiological, and management aspects of LVHT, highlighting the challenging nature of this feature and proposing a practical approach for clinicians.

## 1. Introduction

Left ventricular hypertrabeculation (LVHT), also referred to as left ventricular non-compaction (LVNC) or non-compaction cardiomyopathy (NCC), has undergone shifting interpretations over the past 70 years. This condition was first recognized as a rare ventricular finding during necroscopies, and later gained attention within the cardiology community with the widespread use of echocardiography in the 1980s. As reports of LVHT increased, the molecular basis, genetic background, and clinical implications associated with the condition began to be explored. Meanwhile, conflicting evidence regarding associated complications, as well as the common overlap with other cardiomyopathies, non-cardiac syndromes, and transient conditions, recently led the European Society of Cardiology (ESC) to “downgrade” LVNC to a descriptive phenotype of ventricular myocardial appearance. Meanwhile, the ESC also suggested avoiding the term “LVNC” in favor of “hypertrabeculation.” In this context, we reviewed the historical evolution of LVHT and the associated complications to provide a balanced perspective and a practical management approach on this entity, which continues to attract significant interest within the cardiology community owing to the elusive nature of the condition.

## 2. Origins of “LVHT”

The presence of ventricular muscular bands within the ventricular cavities has been reported since the mid-1970s [1]; however, the first comprehensive anatomical classification, based on necropsy samples, was proposed in 1987 by Boyd *et al*. [2]. The study aimed to characterize ventricular trabeculations, which were increasingly recognized in the early era of echocardiography. In 474 dissected hearts from individuals who died of non-cardiac causes across all age groups, Boyd *et al*. [2] identified 323 cases (68%) of “prominent trabeculation.” This entity was defined as a discrete muscle bundle measuring more than 2 mm in diameter and projecting from the background of the left ventricular endocardium. A slightly higher prevalence of prominent trabeculations was observed in males than in females (72% vs. 65%), with no age-related differences. Regarding trabecular location, 85% originated from the left ventricular wall (LVW) and extended toward the ventricular septum (VS), although muscular bundles connecting two different points of the VS or LVW were also observed. Most trabeculations were oriented from the apex to the mid-segment, with lengths ranging from 2 to 4 cm and diameters of approximately 0.5 cm [2]. In a smaller sample of pathological hearts, Keren *et al*. [3] observed “aberrant bands” and hypertrophic trabeculations in 37% and 43% of left ventricles (LVs) and right ventricles (RVs), respectively. However, more than three trabeculae were rarely present in Boyd’s population of normal hearts [2]; therefore, the presence of more than three trabeculae came to be regarded as the threshold for abnormality.

As echocardiography entered clinical practice, ventricular trabeculae became increasingly detectable by transthoracic echocardiography (TTE), aiding in the differential diagnosis of ventricular thrombi [2,4]. Consequently, TTE became the primary tool for identifying trabeculae.

**Fig. 1.**
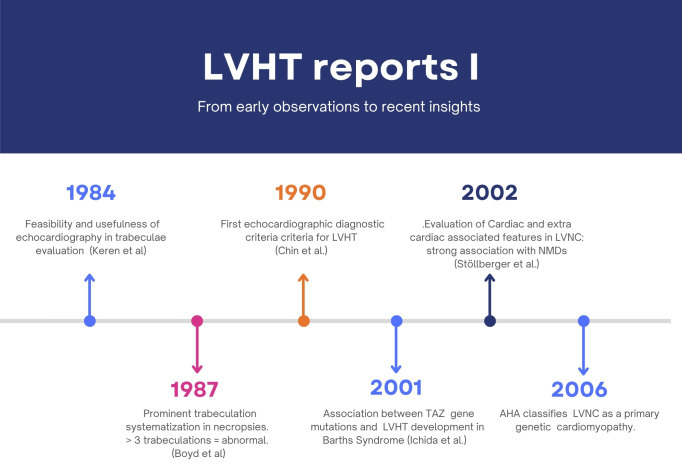
**Timelines of reports I**. LVHT, left ventricular hypertrabeculation; LVNC, left ventricular non-compaction; NMDs, neuromuscular disorders; AHA, American Heart Association.

**Fig. 2.**
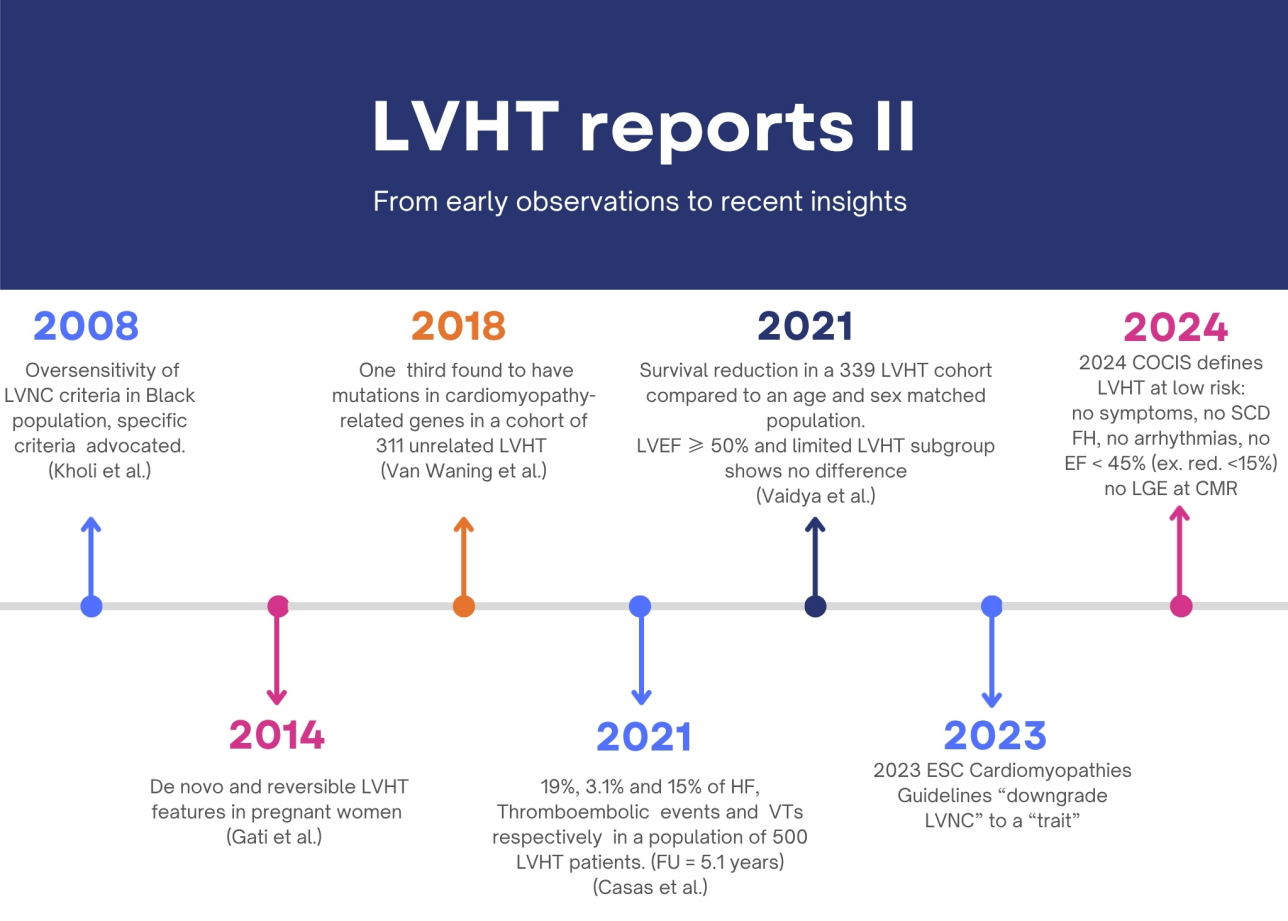
**Timelines of reports II**. LVEF, left ventricular ejection fraction; SCD, sudden cardiac death; FH, family history; LGE, late gadolinium enhancement; CMR, cardiac magnetic resonance; COCIS, Competitive Sport Eligibility in Athletes with Heart Disease; HF, heart failure; ESC, European Society of Cardiology; VTs, ventricular tachycardias; EF, ejection fraction.

Therefore, considerable effort was made to establish a common terminology for describing this trait (Figs. 1,2).

## 3. The Evolution of Diagnostic Criteria

Following the report by Boyd *et al*. [2], the presence of more than three trabeculations in the LV has been considered sufficient for the diagnosis of hypertrabeculation [5,6]. In the late 1980s, the echocardiographic era began, and the first attempt to systematize LVHT diagnostic criteria via ETT was reported [5]. In a small sample of young patients, Chin *et al*. [5] proposed diagnostic criteria based on the presence of excessively prominent trabeculations with deep intertrabecular recesses as the key element, together with a decreasing X/Y ratio from the base to the apex of the LV. The X/Y ratio was defined as the distance from the epicardial surface to the trough of trabeculation (X) divided by the distance from the epicardial surface to the peak of trabeculation (Y). Measurements were taken at end-diastole using both the parasternal long-axis and four-chamber views (Table 1) [5,7,8,9,10,11].

**Table 1.** **Imaging criteria**.

Author (Year)	Modality	Diagnostic criterion	Threshold/description	Population
Chin *et al*. (1990) [5]	Echocardiography	X:Y: the ratio of compacted (X) to non-compacted (Y) myocardium at end-diastole	X/Y ≤0.5 (non-compacted ≥2× compacted)	LVHT with facial dysmorphisms and familial recurrence
Jenni *et al*. (2001) [7]	Echocardiography	A two-layered myocardium with perfused inter trabecular recesses	NC/C ratio >2.1 at end-systole	Isolated LVHT
Stöllberger *et al*. (2013) [8]	Echocardiography	≥3 trabeculations not connected to the papillary muscles	Visible in one plane, both systole and diastole	Unspecified
Petersen *et al*. (2005) [9]	Cardiac MRI	NC/C ratio on short-axis cine MRI at end-diastole	NCC >2.3	LVHT + FH of LVHT or NMDs or WMAs, or thromboembolic events
Jacquier *et al*. (2010) [10]	Cardiac MRI	Non-compacted mass as % of total LV mass	NC mass >20%	Jenni’s criteria fulfilment or borderline criteria + FH
Captur *et al*. (2013) [11]	Cardiac MRI	Trabeculae FD analysis	FD >1.3	LVHT + FH of LVHT or NMDs or WMAs, or thromboembolic events

NC/C, non-compaction/compaction ratio; WMAs, wall motion abnormalities; MRI, magnetic resonance imaging; FD, fractal dimension.

**Fig. 3.**
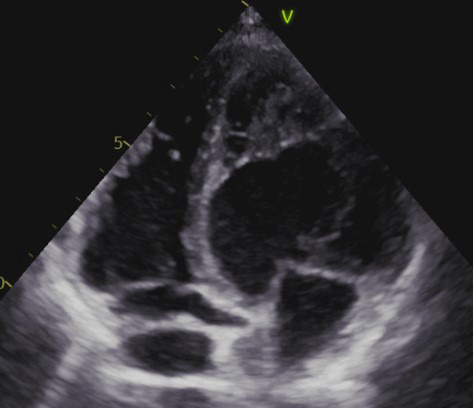
**LVHT appearance in a TTE 4 chamber**.

Using a sample of equivalent size, Jenni *et al*. [7] proposed alternative diagnostic criteria in 2001. The study by Jenni *et al*. [7] examined evidence of a two-layer structure in the hypertrabeculated segments, comprising a compact epicardial layer and an endocardial layer with a prominent trabecular meshwork and deep intertrabecular spaces. This feature was reported to be best visualized in systole, and color Doppler showed typical forward and reversed blood flow within the trabeculations of the ventricular cavity in LVHT cases [7]. By measuring the maximal non-compaction/compaction (NC/C) ratio, the authors found that an end-systolic NC/C ratio >2 was diagnostic of LVHT, thereby allowing unambiguous differentiation from other conditions such as dilated cardiomyopathy (DCM) and left ventricular hypertrophy (LVH) [7]. Non-compaction zones were most frequently observed in the mid-lateral wall, the apex, and the mid-inferior wall, as confirmed by histopathological examination and consistent with previous reports [2]. As the use of cardiac magnetic resonance (CMR) imaging expanded, the authors suggested that other imaging modalities might have been helpful; however, at that time, no CMR diagnostic criteria for LVHT were available [7]. Consequently, “Jenni’s criteria” entered clinical practice and were considered the main ETT criteria in several LVHT studies [12] (Fig. 3).

In 2013, Stöllberger *et al*. [8] proposed new ETT criteria for LVHT. These criteria included the presence of more than three distinct and prominent trabeculations along the endocardial border, moving synchronously and forming the non-compacted layer of a two-layered myocardial structure, best visualized at end-systole [8]. In addition, the demonstration of perfusion of the intertrabecular recesses from the ventricular cavity at end-diastole, assessed by color Doppler or contrast ETT, was introduced as a fourth criterion.

Since standardized measurements of the NC/C ratio were lacking, the authors chose not to consider this metric as reliable [8]. Unfortunately, this consensus of Austrian–German experts did not report the sensitivity or specificity of the proposed LVHT criteria [8]. However, the authors raised important questions, asking whether the number of trabeculations was truly essential for diagnosing LVHT or whether simply identifying a two-layered myocardial structure might be sufficient. Given the uncertainty regarding the number of trabeculae visualized on TTE, the authors stated that applying the historical “>3 trabeculations” cutoff for LVHT [2] might not be appropriate in the TTE setting. Conversely, using recognition of a two-layered structure as the sole criterion may lead to overdiagnosis [8].

**Fig. 4.**
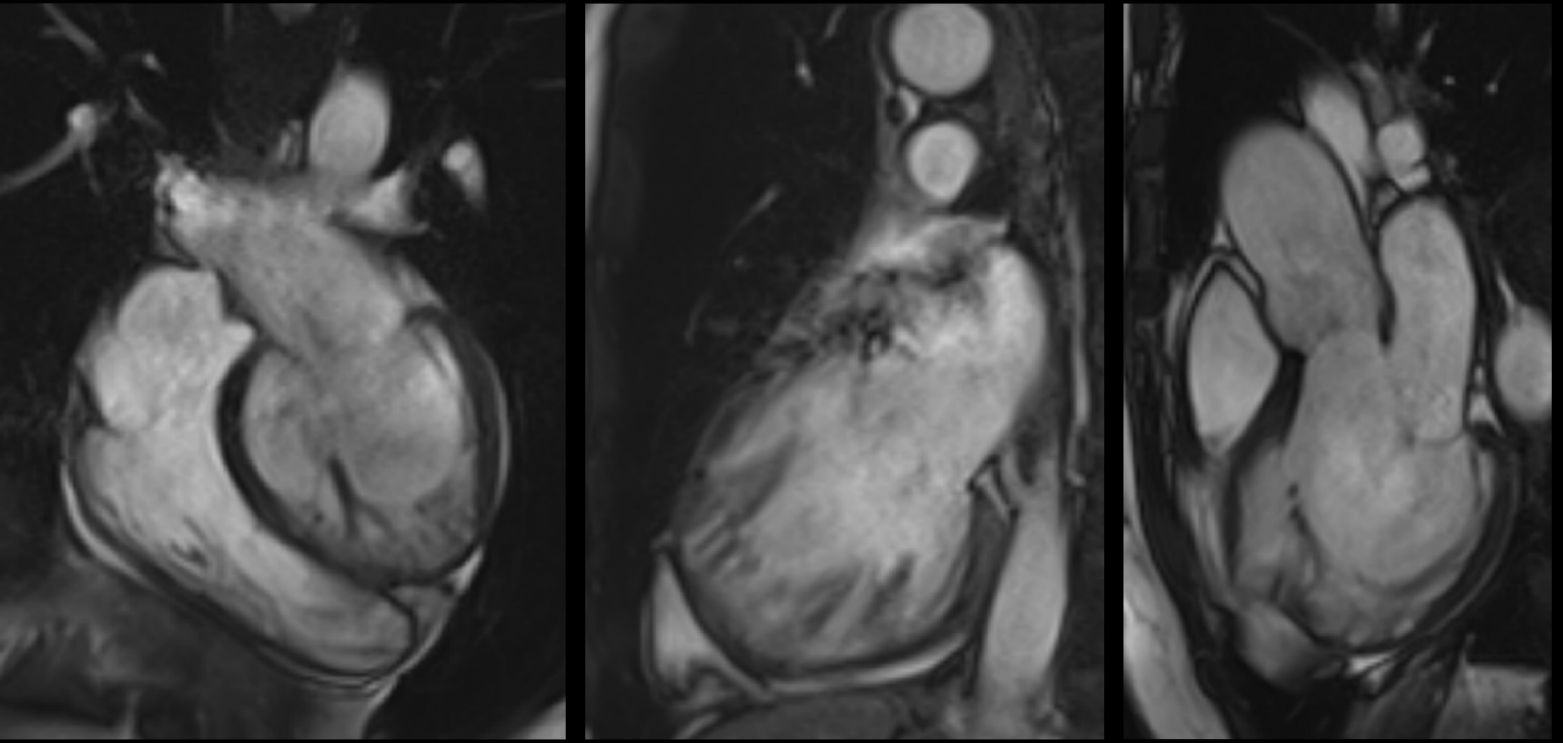
**Steady-State Free Precession cine-CMR images of an LVHT case**.

In 2005, Petersen *et al*. [9] proposed the first CMR criteria for diagnosing LVHT (Fig. 4).

The authors compared a small sample of LVHT cases with healthy volunteers, athletes, patients with hypertrophic cardiomyopathy (HCM), dilated cardiomyopathy (DCM), hypertension-related LVH, and aortic stenosis. Preferential hypertrabeculation zones were similar to those reported previously [7], and a greater number of these zones was observed in the LVHT group [9]. Specifically, the end-diastolic NC/C ratio measured in the short-axis view was 60% higher in the LVHT group, and the receiver operating characteristic (ROC) curve analysis identified an end-diastolic NC/C ratio >2.3 as the most useful cutoff for distinguishing LVHT from the other groups, with a sensitivity of 86% and a specificity of 99%. The positive predictive value (PPV) and negative predictive value (NPV) were 75% and 99%, respectively. Petersen’s work has several limitations. First, the sample size was small, and the high sensitivity of CMR for trabecular detection was not comparable to that of previous TTE-based studies. Additionally, patients with LVHT were enrolled only if the patient had “supportive criteria,” such as an LVHT pattern in first-degree relatives, an association with neuromuscular disorders (NMDs), or complications, such as systemic embolization or wall motion abnormalities (WMAs) [9]; thus, the patient did not represent an isolated LVHT population.

Jacquier’s CMR criteria for LVHT proposed a novel approach [10]. To move away from pathology-based and TTE-derived criteria, the authors focused on trabeculated versus compacted mass and the associated quantification as a possible tool to differentiate LVHT from DCM, HCM, and healthy controls [10]. In the LVHT group, which mostly fulfilled Jenni’s criteria, the trabeculated mass was three times higher, whereas this feature did not change significantly in other groups. ROC analysis showed that a trabeculated mass threshold >20% was associated with LVHT, with specificity and sensitivity of 93.7% each [10].

Using a smart and sophisticated technique, Captur *et al*. [11] proposed new CMR diagnostic criteria in 2013. Given the complexity of the endocardial border, the authors chose to quantify this feature as a continuous variable to reduce the subjectivity associated with previous criteria. The study applied fractal analysis to the endocardial border structure, as previously proposed for other biological applications [13]. This method measures how completely a complex structure fills space, yielding a dimensionless index (fractal dimension (FD)) that ranges from 1 to 2 for the endocardial border [11]. A total of 35 LVHT patients who met Jenni’s criteria and had an additional risk factor were compared with 75 healthy Caucasians and 30 healthy Black individuals. Measurements were obtained in the short axis at end-diastole, and a higher LV FD was observed in the LVHT group than in the other groups, particularly in the apical segments; FD values were higher in Black individuals than in Caucasians. The ROC analysis identified the optimal diagnostic FD thresholds of 1.26 for global LV FD and 1.30 for maximal apical FD, both achieving 100% sensitivity and specificity [11].

In the same year, Stacey *et al*. [14] compared systolic and diastolic methods for assessing isolated non-compaction using CMR. To evaluate the NC/C ratio, the authors focused on the largest NC/C ratio measured in end-diastole and end-systole using the apical short-axis views located 16 to 24 mm from the true apical slice, thereby preventing overestimation of trabeculation that may result from a more apical analysis [14]. The authors found that end-systolic measurements were feasible, and that an end-systolic NC/C ratio ≥2 showed the strongest correlation with heart failure (HF) and clinical events [14].

Currently, several diagnostic methods are proposed by the ESC and the European Association of Cardiovascular Imaging (EACVI) [15]. Jenni’s, Petersen’s, Jacquier’s, Stacey’s, and Captur’s criteria are standard tools to consider when an LVHT diagnosis must be ruled in or ruled out. Since the diagnosis cannot rely exclusively on imaging findings, the ESC/EACVI recommends assessing the pre-test probability, the presence of other cardiomyopathy features, and any history of neuromuscular or developmental anomalies to improve the diagnostic yield of echocardiographic (echo) or CMR findings [15].

This final remark highlights the intrinsic limitations of these criteria (Table 1).

The main issue with these classifications is the population used to develop the proposed criteria. In Chin *et al*. [5], three of eight patients were reported to have facial dysmorphism, and 50% had familial recurrence of LVHT. Jenni *et al*. [7] focused on isolated LVHT, defined as the absence of other cardiac anomalies, whereas Stöllberger *et al*. [8] reported no clinical features in their population. The criteria proposed by Petersen *et al*. [9] were based on only seven LVHT patients, who also had to demonstrate additional features for enrollment, such as a family history of the same trait, associated NMDs, or complications such as thromboembolic events or WMAs; the same additional features were also considered by Captur *et al*. [11]. Jacquier *et al*. [10] focused on 16 LVHT patients, 12 of whom fulfilled Jenni’s criteria, while four were included based on borderline criteria and a positive family history of the same trait. Consequently, the conflict among these criteria is difficult to resolve, and the applicability of these criteria in clinical practice has been disputed [16,17]. In light of the current scientific focus on distinguishing pathological from physiological degrees of ventricular trabeculation, these selection biases appear to be a major reason why the criteria have been downplayed in clinical practice. Cutoff values derived from different, potentially unrepresentative cohorts seem to have limited clinical relevance. Nevertheless, it should be acknowledged that, at the time these criteria were developed, LVHT was considered a distinct cardiomyopathy. Therefore, it is reasonable that different research groups focused on cases of hypertrabeculation with additional cardiac and/or extracardiac findings, whereas only a few addressed isolated hypertrabeculation. To determine whether recognizing and reporting an LVHT phenotype is worthwhile, it is necessary to consider the underlying mechanisms of this appearance and its pathophysiological development.

## 4. Pathophysiology Hypothesis

The architectural development of the myocardium occurs in four stages [18]. During the tubular heart phase, the myocardium exhibits a two-layer epithelial cell structure. At the end of the fourth week, trabeculations begin to emerge. By the eighth week (Carnegie stage 22), further growth occurs, and the radial trabeculae become organized in an apico-basal orientation. Subsequently, along with proliferation and thickening of the myocardial layer, the trabeculae undergo compaction, and the coronary vasculature invades the myocardium in an epicardial-to-endocardial direction [19]. Vascularization of the endocardium and trabeculae gradually shifts from passive blood diffusion in the earlier phases, when trabeculations are also referred to as “sinusoids,” to the final stage where trabeculae become thicker, more prominent in the LV, and nourished by coronary artery branches [18]. A lack of trabeculae formation reduces the oxygen and molecular supply necessary for myocardial function and development, leading to embryonic death. Additionally, the role in the development of the Purkinje system and in the stability of the mitral apparatus has been highlighted previously [20].

The final stage involves multilayering, in which a three-layered spiral system of myocardial architecture is formed, thereby contributing to the compact myocardium [18]. However, an alternative theory suggests that trabecular muscle coalescence is the primary contributor to compaction [21]. Cardiomyocytes that compose the trabeculae have been reported to be more differentiated than those in the compacted zones [18], and cardiomyocytes also appear to be more molecularly mature, with a lower proliferation rate [22]. Different gene expression patterns have also been identified in these two populations [20]. Two important molecular mediators, bone morphogenetic protein 10 (BMP10) and the hairy-related basic helix–loop–helix transcription factor Hey2, have been shown to play crucial roles in cardiomyocyte development and proliferation [20]. Tian *et al*. [21] demonstrated a hybrid zone between the trabecular and compact myocardium in 2017 and, more notably, found that inhibiting Hey2 cells within this zone led to disproportionate, excessive trabeculation.

The hypertrabeculated appearance of LVHT has been associated with arrest of myocardial architectural development after the eighth morphogenetic week [5,23].

Petersen *et al*. [24] recently challenged the longstanding hypothesis that the non-compaction appearance of the myocardium may result from incomplete trabecular compaction during intrauterine development.

To support this paradigm shift, the authors mentioned the independent growth of compacted versus trabecular myocardium [21], proposing that compaction occurs independently of the trabecular portion [24]. The different growth rates of compacted and non-compacted myocardial tissue, referred to as “allometric growth,” were considered the primary factor contributing to the unequal proportions of these myocardial layers [24]. Thus, the authors rejected the term “non-compaction.” However, controversies persist regarding the underlying molecular mechanisms, which remain poorly understood [25,26,27].

## 5. Association With Other Syndromes and Conditions

As previously noted in the development of diagnostic criteria, associated conditions are often reported in LVHT cohorts. Using the presence of more than three trabeculations as a marker of LVHT, as previously demonstrated [2], Stöllberger *et al*. [28] evaluated the association of LVHT with both cardiac and non-cardiac anomalies. Stöllberger *et al*. [28] reported that up to 80% of patients were diagnosed with a NMD during a neurological evaluation.

In 2013, Kimura *et al*. [29] evaluated a cohort of patients with Duchenne muscular dystrophy (DMD) and Becker muscular dystrophy (BMD) and found an LVHT rate of 20%. Interestingly, patients with DMD/BMD presenting with hypertrabeculation showed a higher incidence of left ventricular ejection fraction (LVEF) impairment and a poorer prognosis [29]. The mechanisms underlying the correlation between LVHT and NMDs remain unclear, and no convincing explanation has been reported to date [30].

The contemporary presence of LVHT and Barth syndrome has also been previously reported [31]. This rare X-linked disease is characterized by a mutation in the *TAZ* gene, which encodes the cardiolipin transacylase tafazzin, previously known as G4.5 [32]. In addition to hypertrabeculation, the syndrome may present with specific traits such as myopathy, neutropenia, inadequate growth, prepubertal growth retardation, chronic fatigue, dysmorphic features, and cognitive impairment [32]. Tafazzin deficiency leads to cardiomyopathy by disrupting the formation of respiratory chain supercomplexes, resulting in loss of succinate dehydrogenase in myocardial tissue [32]. This genetic disease may present with an infantile-onset cardiomyopathy, more commonly as DCM [32] or LVHT (50–60%) [33], whereas HCM is less frequent [34].

Rarer syndromes, such as dystrobrevinopathy [35] and Z-band complex anomalies [36], have also been associated with LVHT. However, the genotype–phenotype correlation remains unclear, and published evidence is limited to a small number of case reports [35,36].

One of the main reasons for the recent “downgrading” of the condition formally called LVNC to a “non-cardiomyopathy” in the updated ESC cardiomyopathy classification is the possible *de* *novo* occurrence or increase during transient conditions [37]. For example, LVHT is known to occur in athletes [38], and dedicated diagnostic criteria for this population have previously been proposed [39]. Gati *et al*. [39] found that up to 10% of regional- and national-level athletes met echocardiographic criteria for LVHT. Moreover, high-intensity physical activity in non-competitive settings has also been linked to greater trabeculation [40].

Significant hemodynamic changes occur during pregnancy [41]. Cardiac output, heart rate, and vasodilation increase in the earliest phases; meanwhile, arterial blood pressure decreases, whereas vasomotor response and blood volume rise consistently, and cardiac contractility does not appear to change significantly [41]. Cardiac remodeling has often been reported, with increases in both left and right ventricular mass [42,43]. Regarding LVHT, Gati *et al*. [44] reported increased trabeculation during pregnancy in 25% of a cohort of primigravidae, with a smaller proportion meeting diagnostic criteria for LVHT. Interestingly, nearly two-thirds of these patients showed complete normalization in the postpartum period [44].

An association between LVHT and beta-thalassemia [45] as well as chronic kidney disease [46] has also been reported. However, no molecular or genetic basis for this association has been proposed to date. Indeed, both conditions are characterized by consistent changes in blood volume due to fluid overload and a high-flow state, thus sharing features with pregnancy.

Specific syndromes and transient conditions may present with varying degrees of trabeculation, occasionally meeting the diagnostic criteria for LVHT. Although such associations have been reported, no formal or convincing explanation for these observations has yet been published. Uncertainty stems from small sample sizes, a lack of prospective studies, inconsistencies in the pathophysiologic background, and the limitations and controversy surrounding existing LVHT criteria.

Thicker trabeculations have been reported in Black individuals, although no correlation between ethnicity and NC/C ratio has been found [47]. Kohli *et al*. [6] reported a higher frequency of LVHT in Black individuals than in Caucasians, with LVHT criteria fulfilled across the age spectrum. Consequently, the authors argued that current LVHT criteria may be overly sensitive, particularly in Black patients [6], and suggested that, as with athletes, the Black population may require dedicated LVHT thresholds.

## 6. The Contribution of Genetics and Familial Clustering

The 2006 American Heart Association (AHA) classification of cardiomyopathies considered LVNC a primary genetic cardiomyopathy [48]. The AHA described the isolated form of LVHT as being associated with ZASP (Z-line) and mitochondrial mutations, as well as X-linked or autosomal dominant (AD) inheritance resulting from mutations in the *G4*.*5* gene encoding tafazzin (associated with Barth syndrome) [35,48,49]. Conversely, when LVHT was associated with congenital heart disease (CHD), mutations in α-dystrobrevin and the NKX2.5 transcription factor were considered the main causes [35]. However, these associations were identified in only a few cases, and the reports largely came from familial cases [35,50].

In 2018, van Waning *et al*. [30] retrospectively evaluated 311 unrelated LVHT patients who met Jenni’s or Petersen’s criteria for LVHT and had undergone genetic testing covering 45 cardiomyopathy-related genes. A total of 104 (32%) patients had pathogenic/likely pathogenic mutations, while 111 had variants of unknown significance (VUS). Among gene-positive LVHT patients, 82% had mutations in sarcomere-related genes. *MYH7*, *MYBPC3*, and *TTN* were the most represented (71%), while 11% had mutations in *ACTC1*, *ACTN2*, *MYL2*, *TNNC1*, *TNNT2*, or *TPM1*. Non-sarcomeric mutations were also detected involving *DES*, *DSP*, *FKTN*, *HCN4*, *KCNQ1*, *LAMP2*, *LMNA*, *MIB1*, *NOTCH1*, *PLN*, *RYR2*, *SCN5A*, and *TAZ* [30]. In 2020, Ross *et al*. [51] performed exome sequencing on 35 LVHT patients without a family history of LVHT, including 10 with no associated syndromes or cardiac malformations, using a 193-gene nuclear/mitochondrial panel. The study reported a 9% diagnostic yield for heterozygous pathogenic or likely pathogenic variants in *NKX2*-5 and *TBX5*, which encode cardiac transcription factors, in patients with associated syndromes or cardiac dysfunction. No mutations were reported in isolated LVHT cases. Ross *et al*. [51] concluded that the utility of large-scale genetic screening for non-familial LVHT is limited and that transcription factors should be included in genetic panels.

The literature review and gene curation by Rojanasopondist *et al*. [52] in 2022 represent the largest and most comprehensive evaluation of LVHT genetics to date. The authors reviewed 405 articles reporting genetic data on LVHT and assessed the strength of the association between genetic background and phenotype. The authors identified 189 genes and 19 unique loci associated with chromosomal abnormalities; among these, 11 (6%) were classified as definitive, 21 (11%) as moderate, and 140 (74%) as limited, while 17 (9%) showed no correlation with LVHT [52]. The definitive group comprised *ACTC1*, *DES*, *DSP*, *MIB* *1*, *MYBPC3*, *MYH7*, *RYR2*, *TAZ*, *TPM1*, and *TTN*. A moderate association was found for *ALPK3*, *DMD*, *DMPK*, *DTNA*, *HCN4*, *LAMP2*, *LDB3*, *LMNA*, *NKX2*.*5*, *NNT*, *OBSCN*, *PKP2*, *PLEKHM2*, *PLN*, *PRDM16*, *RBM20*, *SCN5A*, *TBX20*, *TBX5*, *TMEM70*, and *TNNT2*. Genes in the definitive group belonged to several functional domains, including sarcomere function (64%), cellular junction proteins (9%), mitochondrial function (9%), protein degradation (9%), and transcriptional/translational regulation (9%). A comparison of LVHT-related genes with those associated with DCM and HCM identified 24 genes in common. Although the work of Rojanasopondist *et al*. [52] marks an important step forward in understanding LVHT genetics, the study also highlights existing gaps in current knowledge on this disease. The authors clearly state this and question why mutations known to be associated with DCM or HCM would lead to isolated LVHT or a mixed phenotype of LVHT ± DCM/HCM. Although partial explanations may arise from NOTCH pathway alterations or cardiomyocyte polarization, these mechanisms are insufficient to explain the heterogeneity of hypertrabeculation. The authors also proposed epigenetic, environmental, and other non-genetic factors as important contributors [52].

Therefore, current knowledge of LVHT genetics remains limited. Small sample sizes and inconsistent data contribute to an unclear genetic framework, with only sporadic focus on isolated LVHT and many reports involving other syndromes or CHD.

## 7. Epidemiology and Prognosis of LVHT

An adult prevalence of 50 per 100,000 has been estimated for LVHT [53].

The first descriptions of LVHT reported a high risk of reduced LVEF, endocardial thrombosis with systemic embolization, and ventricular arrhythmias [5]. More recent data from a larger population identified these three complications as the most common in patients with LVHT [54]. However, LVEF and late gadolinium enhancement (LGE) on CMR have been reported to be the main prognostic contributors, rather than the degree of trabeculation [55]. Casas *et al*. [55] performed a multicenter retrospective analysis of more than 500 patients with LVHT diagnosed by echocardiography or CMR. During a follow-up of 5.1 years, HF occurred in 110 (19%) patients, whereas systemic embolism and ventricular arrhythmias occurred in 18 (3.1%) and 87 (15%) patients, respectively [54].

Hirono *et al*. [56] reported a thromboembolic prevalence of 6.2% and an annual incidence of 2.9%; similar findings were observed in the meta-analysis by Bhatia *et al*. [57]. Meanwhile, Hirono *et al*. [56] reported a 16% prevalence of ventricular arrhythmias, although higher rates have been reported [26,58,59].

All-cause death occurred in 34 (5.8%) patients in the study by Casas *et al*. [55], with age at diagnosis and male sex identified as predictors of events in the multivariate analysis. The authors highlighted that patients with LVHT who had no ECG abnormalities, no family history (FH), no LGE on CMR, and an LVEF >50% experienced no major adverse cardiovascular events (MACEs) at follow-up.

At a median follow-up of 6.3 years, Vaidya *et al*. [59] evaluated 339 patients with LVHT diagnosed according to Chin’s, Jenni’s, or Petersen’s criteria, and found reduced overall survival in the LVHT group compared with an age- and sex-matched population in the United States. However, patients with an LVEF ≥50% or hypertrabeculation limited to the apical segments showed no difference in survival rate [59]. Other studies comparing LVHT with DCM cohorts reported similar rates of cardiovascular and all-cause mortality, thromboembolic events, and arrhythmias [54]. Evidence on prognosis in LVHT cohorts remains conflicting. Although an increased risk of MACE appears to be recognized, patients with LVHT without LVEF impairment or extensive LGE did not appear to have a higher risk than the general population. Additionally, the prognostic significance of the extent of trabeculation remains uncertain [55].

## 8. Current Recommendations

As the ESC no longer classifies LVHT as a distinct cardiomyopathy, the latest European cardiomyopathy guidelines, published in 2023, do not provide specific recommendations for the management of LVHT [37].

The AHA does not provide comprehensive guidelines for all cardiomyopathies, although the AHA has published cardiomyopathy-specific guidelines [60]. Towbin and Jefferies [53] proposed an approach to LVHT management in 2017. The authors recommended guideline-directed medical therapy (GDMT) for symptomatic HF; meanwhile, the presence of NSVTs was considered sufficient to warrant consideration of an implantable cardioverter-defibrillator (ICD), given the VT rate and the occurrence of appropriate ICD therapies at follow-up [61]. When appropriate, heart transplantation or left ventricular assist devices were also considered possible strategies. Regarding GDMT and the prevention of sudden cardiac death (SCD), the ESC guidelines for HF and SCD consider LVHT a subtype of DCM; therefore, these guidelines apply the same treatment options and ICD implantation recommendations [62,63].

Regarding the anticoagulation treatment, Towbin and Jefferies [53] deemed it necessary in appropriate clinical settings, such as concomitant atrial fibrillation, following a thromboembolic event, or when atrial or ventricular thrombi are identified. Vitamin K antagonists (VKAs) are the preferred agents in this clinical scenario, as data on direct oral anticoagulants (DOACs) are lacking. The 2019 Heart Rhythm Society (HRS) expert consensus statement on arrhythmogenic cardiomyopathy reported that anticoagulation was recommended in individuals with LVHT and atrial fibrillation, as well as in those with previous embolic events (Class of recommendations (CoRs) = 1; level of evidence (LoE) = B); anticoagulation was also considered reasonable in individuals with LVHT and evidence of ventricular dysfunction (CoR = IIB; LoE = B). No specific recommendations were reported regarding arrhythmia management.

In 2024, the Italian Cardiological Guidelines for Competitive Sport Eligibility in Athletes with Heart Disease (COCIS) were updated [64]. The document highlights the importance of distinguishing mild LVHT, commonly seen in athletes, from true LVHT cases. In confirmed LVHT cases, individuals were classified as low risk if none of the following factors were present: symptoms (particularly syncope), family history of SCD, significant atrial or ventricular arrhythmias, LV dysfunction (EF <45% with exercise reduction <15%), or LGE on CMR [64].

## 9. Discussion

LVHT, currently the preferred term for LVNC and also referred to as NCC, is a complex and multifaceted “trait.” Traditionally regarded as a congenital cardiomyopathy with a defined genetic background, LVHT is now increasingly considered an anatomical variant. Given that the available diagnostic criteria cannot distinguish isolated LVHT from LVHT associated with other features, that the genetic basis of isolated forms appears weak, and that studies have identified spurious LVHT cohorts, it is understandable why this condition has progressively lost its status as a cardiomyopathy over time.

However, because LVHT remains incompletely understood, it may be overly simplistic to reduce such a substantial body of evidence to a mere “trait,” suggesting that it may or may not warrant reporting during routine TTE. Several key points emerge from the data gathered in our review. First, the diagnostic criteria for LVHT should be regarded as tools for identifying an abnormal degree of ventricular trabeculation rather than for diagnosing a disease. In this sense, the criteria may be better viewed as a grading system that helps clinicians define a threshold for excessive trabeculation.

The pathophysiological mechanisms underlying LVHT remain poorly understood. Although some progress has been made in recent years, current knowledge remains insufficient to conclude that this “anatomical variant” has been fully characterized. In isolated forms, the genetic background does not appear to meaningfully affect patient management; however, in familial cases and in those associated with other cardiac or extracardiac abnormalities, genetic evaluation is essential for both diagnosis and management (*e*.*g*., Barth syndrome).

Transient conditions may trigger or exacerbate trabeculation; therefore, a patient may meet LVHT criteria at one time point but no longer do so once the external trigger has resolved. Importantly, LVHT is not a uniform condition. Several high-quality studies have clearly demonstrated prognostic implications associated with LVHT, including a higher prevalence of HF, arrhythmias, and thromboembolic events. The main challenge in LVHT management is identifying the subset of patients who will develop left ventricular dysfunction, arrhythmias, or embolic events.

Although LVHT does not conform to the classical definition of cardiomyopathy, the ESC guideline approach, intended to avoid overdiagnosis and unnecessary concern regarding this “trait,” may appear somewhat dismissive.

In our opinion, a more tailored approach should be proposed. When LVHT is suspected, especially on TTE, CMR should be performed to confirm the diagnosis. If the patient meets the TTE and CMR criteria, high-risk features should be assessed and excluded, including family history of LVHT (with genetic testing considered in familial clusters), LGE on CMR, arrhythmias, and impaired LVEF. If these high-risk factors are absent, reassessment every 3–4 years may be sufficient to monitor for complications, such as arrhythmias, LV thrombus, and LVEF impairment. If high-risk factors are present, annual follow-up should be recommended to help prevent complications and guide therapeutic decisions when appropriate.

In summary, this “trait” should be considered a risk factor for the development of left ventricular dysfunction, arrhythmias, and thromboembolic events. The focus of the clinician should be on identifying cases that progress from isolated mild LVHT to overt cardiomyopathy and on understanding the clinical course of the high-risk subset, while avoiding overtreatment in patients with milder forms.

## 10. Conclusions

LVHT, also referred to as LVNC or NCC, has become increasingly recognized with the widespread use of echocardiography. Although LVHT does not fully meet the traditional criteria for cardiomyopathy, the clinical significance of the condition remains heterogeneous. While many patients experience a favorable course, a subset may develop left ventricular dysfunction, arrhythmias, and thromboembolic events.

Current diagnostic criteria mainly identify an abnormal degree of trabeculation rather than a distinct disease, and genetic and molecular data remain limited, particularly in isolated forms. In this context, a risk-based approach may represent a reasonable strategy. Patients without high-risk features often have good outcomes and may benefit from periodic follow-up, whereas LVHT cases with additional clinical, functional, or imaging abnormalities may require closer surveillance. Therefore, a tailored, cautious, and individualized clinical approach appears appropriate for LVHT in clinical practice.
